# The Inhibition of *Streptococcus mutans* Biofilms following Exposure to Different Chocolate Ingredients

**DOI:** 10.1055/s-0045-1809027

**Published:** 2025-05-06

**Authors:** Hadi A. Almoabid, Leen Saleh Almutairi, Abdul Samad Khan, Mohammed A. Aljaffary, Rasha AlSheikh, Khalid S. Almulhim, Abdulrahman A. Balhaddad

**Affiliations:** 1 College of Dentistry, Imam Abdulrahman Bin Faisal University, Dammam, Saudi Arabia; 2 Department of Restorative Dental Sciences, College of Dentistry, Imam Abdulrahman Bin Faisal University, Dammam, Saudi Arabia

**Keywords:** antibacterial, biofilms, caries, chocolate, prevention

## Abstract

**Objectives:**

This project aimed to investigate the anticariogenic effect of four chocolate ingredients (polyphenol, theobromine, cacao, and flavanol) against *Streptococcus mutans* biofilms grown *in vitro* .

**Materials and Methods:**

Stored *S. mutans* (UA 159) was transferred to Brain Heart Infusion (BHI) broth and incubated in aerobic incubator for 24 hours at 37°C in 5% CO _2_ . Following this, 190 µL of each ingredient concentration (0.78–200 mg/mL) and 10 µL of the culture were added to a 96-well plate and incubated for 24 hours at 37°C in 5% CO _2_ . Then, biofilms were fixed, stained with crystal violet, and analyzed for formation using a spectrophotometer. Control groups included negative control with only *S. mutans* and sterility control with BHI media.

**Statistical Analysis:**

One-way analysis of variance and Tukey tests analyzed the data.

**Results:**

Flavonoid at the 6.25 to 25 mg/mL concentrations reduced the *S. mutans* biofilms ( *p* < 0.001) by 5- to 33-fold. Meanwhile, 50 mg/mL concentrations and higher completely eradicated biofilm growth. Similarly, cocoa concentrations ranging between 12.5 and 200 mg/mL revealed massive antibiofilm action from a 22-fold reduction at 12.5 mg/mL to complete biofilm eradication at 200 mg/mL. Polyphenol was the only ingredient showing biofilm inhibition at all concentrations ranging from almost 10-fold reduction to complete biofilm eradication, which were all significant ( *p* < 0.001) compared to the control. Regarding theobromine, 3.125 mg/mL of it significantly increased the growth of *S. mutans* biofilms. At the concentration of 6.25 mg/mL, theobromine significantly ( *p* < 0.001) inhibited the *S. mutans* biofilms by 3.35-fold. While at the range of 25 to 200 mg/mL, theobromine resulted in a reduction between 11-fold and complete biofilm eradication.

**Conclusion:**

The findings suggest that flavonoid, cacao, polyphenol, and theobromine may serve as effective adjuncts in preventing dental caries by inhibiting *S. mutans* biofilm formation.

## Introduction

Dental caries is a multifactorial chronic disease that causes teeth demineralization and destruction. 1 It mainly occurs due to the microbial community imbalance inside the oral environment due to the lack of oral hygiene measures, where the increased pathogenicity of caries-related pathogens allows the utilization of the available carbohydrates to produce lactic acid and attach hard dental tissues. 2 The rise in consumption of sugary and processed foods and poor oral hygiene practices have contributed to the increase in dental caries incidence, particularly among vulnerable populations, such as children and low-income communities. 3 Therefore, implementing more approaches to control the onset of dental caries is very important to reduce the burden on individuals and health care systems. 4

Recently, light has been shed on using plant-derived and natural compounds and their properties as potential alternatives for traditional synthetic antimicrobial compounds. 5 6 These natural compounds regularly demonstrate varied action mechanisms, such as restricting cellular metabolism, interrupting bacterial cell membranes, and restraining virulence factors. 5 6 Due to their lower toxicity, compatibility, and reduced risk of resistance development, these natural and plant-based antibacterial agents provide numerous benefits when developing therapeutic compounds to target biofilm-triggered diseases. 7 8 As the global concern over antibiotic resistance continues to grow, the investigation and utilization of natural antibacterial compounds from plants and other natural sources have become an active area of research, with the potential to design unique, biocompatible, and effective antimicrobial platforms. 7 8

Individuals of different ages and backgrounds consume chocolate widely due to its assumed delicious taste. Despite the common assumption that chocolate has high carcinogenicity, some scientific evidence suggests that certain ingredients may possess antibacterial properties. 9 Literature stated that theobromine, one of the chocolate ingredients, has an inhibitory action against bacterial enzymes and subsequently inhibits bacterial growth. 10 11 Others found that flavanol may interfere with bacterial adhesion, making it a potential compound to prevent biofilm growth. 10 11 Recent investigations have shown that a mouthwash containing cocoa husk reduced the count of pathogens inside the oral cavity. 12 All these observations suggest that chocolate ingredients could be potential compounds to target oral pathogens and improve oral hygiene measures.

While laboratory studies provide insights into the potential antibacterial effects of chocolate ingredients, more research is needed to determine their effectiveness against caries-related pathogens, mainly *Streptococcus mutans* , and to explore the efficacy of other chocolate compounds. The processing of chocolate during manufacturing, as well as the added flavoring ingredients like milk, sugar, and others, dilute the anticariogenic ability of the chocolate by reducing the concentration of the bioactive compounds. 13 14 Therefore, this project aimed to investigate the anticariogenic effect of four chocolate ingredients against *S. mutans* biofilms grown *in vitro* . It was hypothesized that chocolate ingredients (polyphenol, theobromine, cacao, and flavanol) would inhibit the growth of *S. mutans* biofilms.

## Materials and Methods

### Sample Size Calculation and Study Design

Based on prior studies, 5 6 15 the within-group standard deviation of the absorbance measurements for biofilm formation was estimated to be 0.15. Thus, this study had an 80% power to detect a difference at a 5% significance level, with three to four samples in each of three repeated experiments, resulting in 9 to 12 samples per group. Each chocolate ingredient (flavonoid, cacao, polyphenol, and theobromine) was mixed with Brain Heart Infusion Broth (BHIB) supplemented with 2% of sucrose at the following concentrations: 200, 100, 50, 25, 12.5, 6.25, 3.125, 1.56, and 0.78 mg/mL and each concentration represented a subgroup resulting in a final number 36 experimental group. A group with no ingredients was used as a control. The source of the four ingredients and their manufacturers' details are illustrated in Table 1 .

**Table 1:** The chocolate ingredients used in the study with their source and composition

Ingredient	Manufacturer	Composition
Flavonoid (90 g)	Eclectic herb, Gresham, OR, USA	Dietary fiber, 1 g Sugars, 1 g Vitamin C (ascorbic acid), 2 mg Iron, 0.5 mg Flavonoids (quercetin), 5 mg
Cacao (340 g)	NOW Foods, Bloomingdale, IL, USA	Total fat, 0.5 g Total carbohydrate, 1 g
Polyphenol (250 g)	Bulk Supplements, Henderson, NV, USA	Green tea extract ( *Camellia sinensis* ) Standardized to contain > 50% polyphenols
Theobromine (100 g)	Bulk Supplements, Henderson, NV, USA	−

### Effect of Chocolate Ingredients Supplements on *S. mutans* Growth

**Fig. 1:**
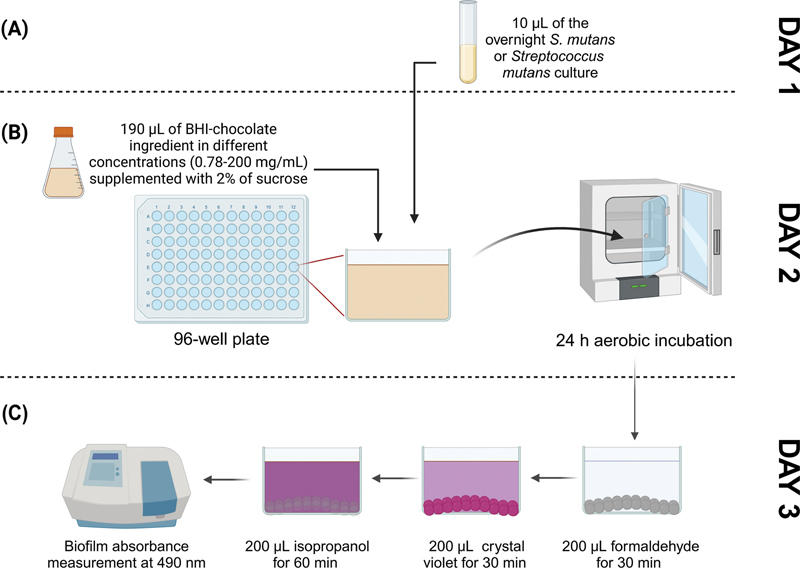
A schematic drawing illustrating the design of the study. ( **A** ) On day 1, *Streptococcus mutans* was grown overnight in 5 mL of brain heart infusion (BHI). ( **B** ) On day 2, 190 µL of each ingredient concentration was incubated with 10 µL of the overnight culture for another 24 hours. ( **C** ) The planktonic growth was discarded on day 3, and the biofilms were fixed, stained, and measured at 490 nm using a spectrophotometer.

The schematic draw for the methodology of this study is shown in Fig. 1 . First, stored *S. mutans* UA 159 (ATCC 700610, American Type Culture Collection, Rockville, Maryland, United States) was transferred to 5 mL of BHIB and incubated in aerobic incubator for 24 hours at 37°C in 5% CO _2_ . The following day, 190 µL of each ingredient concentration was placed in a sterile 96-well flat-bottom microtiter plate. Then, 10 µL of the overnight culture of *S. mutans* (approximately 10 ^6^ colony-forming units/mL) were added to each well. The plates were incubated for 24 hours at 37°C in 5% CO _2_ . The following day, the total absorbance of the culture (including planktonic cells and biofilm) was measured at 590 nm (SpectraMax M5, Molecular Devices, Sunnyvale, California, United States).

To fix the biofilm cells, 200 µL of 10% formaldehyde was added to each well and incubated for 30 minutes. The biofilms were then washed three times with deionized water. Next, 200 µL of 0.5% crystal violet dye was added to each well and incubated for 30 minutes to stain the biofilm. The biofilm cells were washed three times with deionized water. To extract the crystal violet, 200 µL of 2-isopropanol was added to each well and incubated for 1 hour. Finally, the biofilm formation was measured using a spectrophotometer at 490 nm. 5 6 15 The study included two control groups: a negative control consisting of only *S. mutans* overnight culture and BHIB supplemented with 2% sucrose and a sterility control group with only BHIB growth media to ensure no microbial contamination.

### Statistical Analysis

All data were presented as the means ± standard deviations derived from a minimum of three biological replicates. Data normality and distribution were checked using the Shapiro–Wilk test. One-way analysis of variance and Tukey tests were utilized to compare the effects of flavonoid, cacao, polyphenol, and theobromine on biofilm and total growth (Sigma Plot 12.0; SYSTAT). A *p* -value of < 0.05 was considered statistically significant.

## Results

**Fig. 2:**
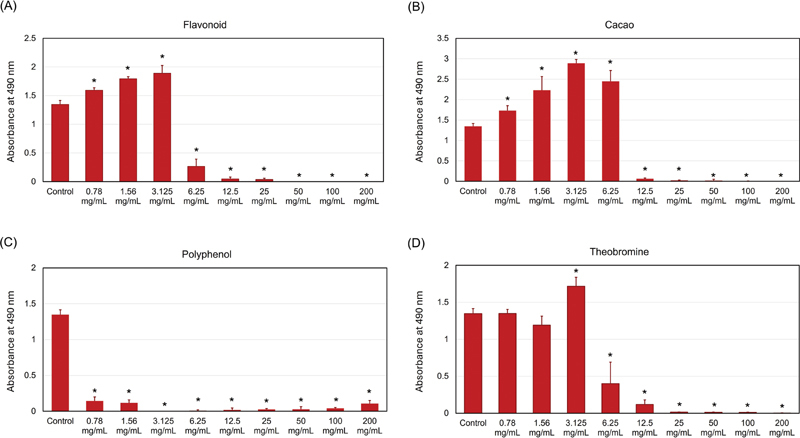
The antibiofilm effect of different chocolate ingredients against *Streptococcus mutans* . ( **A** ) Flavonoid, ( **B** ) cacao, ( **C** ) polyphenol, and ( **D** ) theobromine significantly inhibited the *S. mutans* biofilm. Asterisks indicate a significant difference from the control following the one-way analysis of variance (ANOVA) analysis ( *p* ≤ 0.05).

Fig. 2 illustrates the antibacterial effect of the four chocolate ingredients against *S. mutans* biofilms. In Fig. 2A , flavonoids at 6.25 mg/mL concentrations significantly reduced the *S. mutans* biofilms ( *p* ≤ 0.001) by fivefold. Meanwhile, 12.5 and 25 mg/mL flavonoid concentrations reduced the *S. mutans* biofilm growth by around 33-fold. Concentrations of 50 mg/mL result in a complete eradication of the *S. mutans* biofilms, while flavonoids at concentrations of 0.78 and 1.56. Moreover, 3.125 mg/mL significantly ( *p* ≤ 0.001) increased the *S. mutans* biofilm. Similar observations were observed when cacao was incubated with the *S. mutans* culture ( Fig. 2B ). Concentrations ranging between 0.78 and 6.25 significantly ( *p* ≤ 0.001) increased the biofilm growth. Concentrations between 12.5 and 200 mg/mL revealed massive antibiofilm action from a 22-fold reduction at 12.5 mg/mL to complete biofilm eradication at 200 mg/mL.

Polyphenol was the only ingredient showing biofilm inhibition at all concentrations ( Fig. 2C ), ranging from almost 10-fold reduction to complete biofilm eradication, which was all significant ( *p* ≤ 0.001) compared to the control with no polyphenol. Lastly, Fig. 2D illustrates the antibiofilm action of theobromine. There was no significant difference compared to the control when the biofilm was treated with 0.78 and 1.56 mg/mL of theobromine, while 3.125 mg/mL of it significantly increased the growth of *S. mutans* biofilms. At the concentration of 6.25 mg/mL, theobromine significantly ( *p* ≤ 0.001) inhibited the *S. mutans* biofilms by 3.35-fold, while at the range of 25 to 200 mg/mL, theobromine resulted in a reduction between 11-fold and complete biofilm eradication.

**Fig. 3:**
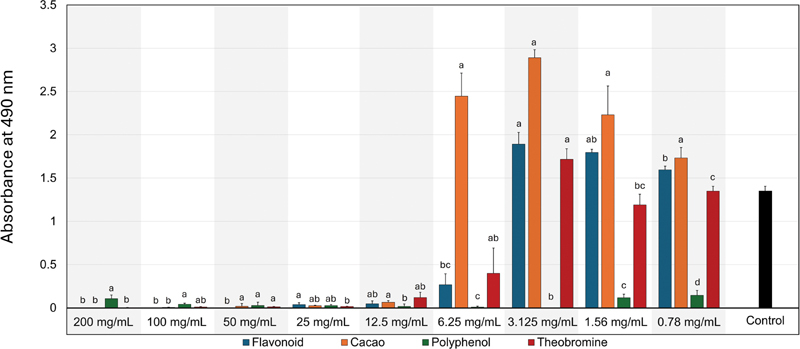
Using one-way analysis of variance (ANOVA), a comparison between the chocolate ingredients (flavonoid, cacao, polyphenol, and theobromine) was conducted at each concentration. Different letters represent significant differences ( *p* ≤ 0.05).

Fig. 3 illustrates the comparison between the four ingredients at each specific concentration. For example, at the 200 mg/mL concentration, all the ingredients revealed significant ( *p* ≤ 0.001) biofilm reduction compared to the polyphenol. At the concentrations of 12.5 to 100 mg/mL, all the ingredients were comparable to each other despite the statistical difference. At low concentrations ranging between 0.78 and 6.25 mg/mL, polyphenol revealed the greatest antibiofilm actions among the investigated ingredients.

## Discussion

Several natural and plant-derived compounds represent potential therapeutic approaches to target biofilm-triggered diseases and associated pathogens. In this study, four chocolate ingredients revealed their capability to inhibit the growth of *S. mutans* biofilms, so the hypothesis was accepted. The findings of this study suggest that effectively utilizing these compounds may inhibit the growth of caries-related pathogens, opening new avenues in caries prevention. Chocolates produced by various industries and found in different markets are typically modified and processed to contain high levels of sugar. 16 17 This high sugar content makes chocolate a highly cariogenic product, despite the presence of other beneficial compounds. 11 12 13 Therefore, this study focused on investigating the effectiveness of these compounds in their raw form against cariogenic biofilms.

*S. mutans* has been recognized as one of the primary key pathogens in the formation and progression of dental caries. It is well-known that microbes in their biofilm forms are more pathogenic than their planktonic counterpart. 18 19 Microbial biofilms are more challenging to eliminate and can prevent the diffusion of therapeutic compounds by forming a polysaccharide matrix. 20 21 In the biofilm forms, *S. mutans* can produce acid to demineralize the tooth structure and sustain their activity despite the high acidity in the environment. 22 Considering all these challenges related to microbial biofilms, it is critical to apply this microbial form while assessing the antimicrobial properties of therapeutic compounds.

Among the ingredients studied, polyphenol showed the strongest antibiofilm action, as all concentrations significantly reduced the growth of *S. mutans* biofilms. In contrast, the other ingredients, flavonoid, cacao, and theobromine, were effective in inhibiting biofilm growth only at concentrations above 3.125 to 6.25 mg/mL. These findings agree with several previous studies, which studied the effect of the tested ingredients separately. 23 24 25 26 Moreover, polyphenol was documented to have a potent effect against *Candida albicans* and have a favorable effect as an antioxidant. 27 It is known that there is a synergetic relation between *C. albicans* and *S. mutans* , which increases the potential of the microorganism to create biofilm both *in vitro* and *in vivo* . 28 Accordingly, regardless of its source (cocoa, green tea, cranberry), polyphenol-rich sources offer a promising natural approach to reducing the *S. mutans* population in the oral cavity, highlighting their potential role in preventive dental care. 24 It is reported that the phenolic compounds act at a cellular level, where cell membrane permeability changes occur. This could be induced by the hydrogen binding of phenolic compounds to enzymes. 29 Increasing the lipophilic nature of phenolic compounds boosts their antimicrobial activity by promoting interaction with the cell membrane, thereby inhibiting intracellular enzymes. 30

Flavonoids are known to have wide-ranging antibacterial activities with antimicrobial effects on fungi, Gram-negative, and Gram-positive bacteria. 25 Naturally available compounds such as flavonoids in food like fruits, vegetables, tea, and coca display antibacterial properties, impairing *S. mutans* metabolism and reducing acid production by the primary cariogenic pathogen. 22 31 Additionally, studies showed that flavonoids could inhibit the ability of *S. mutans* to attach to dental surfaces by disrupting the bacterial membrane, effectively limiting plaque buildup and lowering the caries risk. 22 31 In the current study, flavonoids showed a significant reduction of *S. mutans* biofilms at the concentrations of 6.25 mg/mL *S. mutans* biofilms by fivefold. Additionally, 12.5 and 25 mg/mL flavonoid concentrations reduced the *S. mutans* biofilm growth by around 33-fold. Concentrations of 50 g/mL result in a complete eradication of the *S. mutans* biofilms. These findings were comparable with some of the previous studies 32 33 34 35 in which the flavonoid ingredients, regardless of their type or source (sweet orange waste, guaijaverin, phloretin, grape seed extract), showed a significant reduction of *S. mutans* biofilm. It is anticipated that the flavonoids may inhibit energy metabolism and deoxyribonucleic acid synthesis, thus affecting protein and ribonucleic acid synthesis. 36

Saha et al 33 reported a significant effect after using fewer flavonoid concentrations (2 mg/mL) when extracted from sweet orange waste. This could be explained by the different sources and extraction methods performed (microwave-assisted extraction) that induce altered effects. In alignment with that, Castellanos et al 32 systematically reviewed the available scientific evidence from *in vitro* studies regarding the effect of flavonoids extracted from grape seeds and cranberries on reducing *S. mutans* biofilm. It was found that, in most of the reviewed articles, the significant biofilm reduction can start at low concentrations (0.5, 1, 2 mg/mL) of flavonoids extracted from grape seeds and cranberries.

One of the tested ingredients, theobromine, is a bitter alkaloid found in various fruits, including cocoa, and has shown promising antibacterial properties, particularly against *S. mutans* . Theobromine is naturally present in cocoa powder, ranging from 1.2 to 2.4% in mild chocolate and with a higher concentration in dark chocolate. 37 Studies have also explored the incorporation of theobromine into restorative dental materials to enhance their therapeutic properties. 38 The bacteriostatic effect of theobromine has been evaluated in glass ionome (GIC), demonstrating its ability to inhibit bacterial proliferation. Based on previous results by Cevallos González et al, 38 a 1% theobromine addition to GIC can simultaneously achieve the antibacterial effect and improve the material's microhardness. Additionally, when theobromine was added to a commercial toothpaste, it was found to show the highest efficacy on *S. mutans* compared to the other ingredients tested. 23 Another study by Amaechi et al reported that 0.0011 mol/L of theobromine in artificial saliva improved the remineralization capabilities of demineralized enamel. 39 These findings suggest that incorporating theobromine into toothpastes and mouthwashes could be effective to prevent dental caries and tooth demineralization.

Similarly, the present study tested the antibiofilm action of theobromine, and it was found that theobromine started to significantly inhibit the growth of *S. mutans* biofilms at the concentration of 6.25 mg/mL. While at the range of 25 to 200 mg/mL, theobromine resulted in a reduction between 11-fold and complete biofilm eradication. In addition to its antibacterial activity, theobromine has demonstrated remineralizing potential by promoting the formation of hydroxyapatite crystals on enamel surfaces. In an *in vivo* study, 40 a significant increase in remineralization potential in the tested primary teeth was observed when different treatment modalities were introduced, among which was theobromine-containing toothpaste, which significantly enhanced the resistance of the enamel crystals to subsequent acid challenges.

While chocolate is believed to be one of the primary causes of dental carious lesions, this is a common misconception. Chocolate derived from cocoa beans does not rank highest among cariogenic substances. 37 Particular cocoa plant components can be beneficial in caries prevention. For example, the cocoa butter in chocolate provides a protective layer over teeth, reducing damage caused by bacterial byproducts and acid attacks. 41 Research has indicated that cocoa bean husk extracts can be effective as mouth rinses, demonstrating better antiplaque efficacy compared to traditional mouthwashes, thus supporting their role in oral health. 42 In the current study, cacao concentrations ranging between 12.5 and 200 mg/mL revealed massive antibiofilm action. Incorporating polyphenols, flavonoids, theobromine, and cacao into oral health products like mouthwash and toothpaste may present a promising strategy for controlling dental caries and plaque formation. By formulating oral care products that leverage these natural compounds, manufacturers can enhance their efficacy in promoting oral health and reducing the incidence of dental caries, providing consumers with a more effective and natural approach to dental hygiene. The compounds investigated in this study could be incorporated into toothpastes, mouthwashes, varnishes, and various oral care products as natural and organic strategies for preventing caries. 43 44 This approach may help reduce the reliance on fluoride-containing products for children who are at risk of fluoride ingestion, as well as on chlorhexidine, which is not recommended for long-term use.

The potential of combining natural anticariogenic compounds with different treatment modalities is an exciting and promising aspect of future research that can be explored. However, one limitation of the current study is that each ingredient was tested individually. A thing to consider to fully understand the component's anticariogenic potentials is the effect these compounds might have on one another when combined in one product, as they are naturally present in cocoa beans; the interactions between the ingredients may alter or enhance their properties. Also, this research primarily investigated the antibacterial effects of chocolate ingredients on *S. mutans* . It is important to acknowledge that various other pathogens present in the oral microbiome also play critical roles in maintaining oral health. Future studies should aim to assess the impact of chocolate ingredients on a broader spectrum of dental pathogens to develop a more thorough understanding of their antibacterial properties. Additionally, this study did not take into account the potential influence of different environmental factors that could affect the antibacterial efficacy of chocolate ingredients. Utilizing clinical translation models will be essential for evaluating the effectiveness of chocolate ingredients in the actual oral environments and for exploring their potential incorporation into dental care practices.

## Conclusion

This study demonstrates the significant antibacterial effects of various chocolate ingredients against *S. mutans* biofilms. Overall, polyphenols emerged as the most effective ingredient at lower concentrations, while flavonoids, theobromine, and cacao provided robust antibacterial effects at higher concentrations. These findings suggest that chocolate ingredients, particularly polyphenols, could be beneficial in developing strategies to combat *S. mutans* biofilms, potentially aiding in dental health management.
